# Will Patients Benefit from Regionalization of Gynecologic Cancer Care?

**DOI:** 10.1371/journal.pone.0004049

**Published:** 2009-01-06

**Authors:** Kathleen F. Brookfield, Michael C. Cheung, Relin Yang, Margaret M. Byrne, Leonidas G. Koniaris

**Affiliations:** 1 Department of Obstetrics and Gynecology, University of Miami Miller School of Medicine, Miami, Florida, United States of America; 2 DeWitt Daughtry Family Department of Surgery, University of Miami Miller School of Medicine, Miami, Florida, United States of America; 3 Department of Epidemiology and Public Health, University of Miami Miller School of Medicine, Miami, Florida, United States of America; Tulane University, United States of America

## Abstract

**Objective:**

Patient chances for cure and palliation for a variety of malignancies may be greatly affected by the care provided by a treating hospital. We sought to determine the effect of volume and teaching status on patient outcomes for five gynecologic malignancies: endometrial, cervical, ovarian and vulvar carcinoma and uterine sarcoma.

**Methods:**

The Florida Cancer Data System dataset was queried for all patients undergoing treatment for gynecologic cancers from 1990–2000.

**Results:**

Overall, 48,981 patients with gynecologic malignancies were identified. Endometrial tumors were the most common, representing 43.2% of the entire cohort, followed by ovarian cancer (30.9%), cervical cancer (20.8%), vulvar cancer (4.6%), and uterine sarcoma (0.5%). By univariate analysis, although patients treated at high volume centers (HVC) were significantly younger, they benefited from an improved short-term (30-day and/or 90-day) survival for cervical, ovarian and endometrial cancers. Multivariate analysis (MVA), however, failed to demonstrate significant survival benefit for gynecologic cancer patients treated at teaching facilities (TF) or HVC. Significant prognostic factors at presentation by MVA were age over 65 (HR = 2.6, p<0.01), African-American race (HR = 1.36, p<0.01), and advanced stage (regional HR = 2.08, p<0.01; advanced HR = 3.82, p<0.01, respectively). Surgery and use of chemotherapy were each significantly associated with improved survival.

**Conclusion:**

No difference in patient survival was observed for any gynecologic malignancy based upon treating hospital teaching or volume status. Although instances of improved outcomes may occur, overall further regionalization would not appear to significantly improve patient survival.

## Introduction

To date, studies on the relationship between survival and hospital volume and teaching status for gynecologic malignancies have focused primarily on ovarian cancer.[1], [2], [3], [4], [5], [6], [7], [8], [9], [10] A systematic review of the effect of specialized care for ovarian cancer patients found no demonstrable benefit by surgeon specialty for earlier stages of disease, but that surgery by a gynecologic oncologist resulted in a 5 to 8 month median survival benefit for patients with advanced stage disease.[9], [11] Similar findings have been reported by studies conducted outside the United States.^2^ More recently, however, data from the National Cancer Institutes (NCI) Surveillance Epidemiology and End Results (SEER)-linked Medicare database suggests no benefit for ovarian cancer treated by gynecologists versus gynecological oncologist.[12] As well, SEER-linked Medicare has been used to demonstrate similar outcomes for the use of chemotherapy when administered by medical oncologists or gynecologic oncologists.[13]

Similar to the more recent studies in ovarian cancer, studies investigating optimal treatment paradigms for early-stage endometrial cancer have found minimal differences in outcomes between general gynecologists and gynecologic oncologists.[14], [15] Studies on advanced stage endometrial cancer patients, focused on hospital volume, have suggested a potential survival benefit for certain subsets of patients, such as the elderly, treated at high-volume centers.[16], [17] To date, the effects of center volume or teaching status on outcomes of patient with cervical or vulvar cancers or uterine sarcomas have not been reported in the literature.

In contrast to SEER-linked Medicare data, treatment information, including chemotherapy, can be determined for all patients by treating facility in the Florida Cancer Data System (FCDS), which is well validated in determining outcome disparities and treatment differences by center volume or teaching status.[18], [19], [20], [21], [22], [23] We therefore examined the FCDS to provide insight into the entire field of gynecological oncology by examination the major gynecologic malignancies - cervical, ovarian, endometrial, uterine sarcoma, and vulvar cancers - with the hope of evaluating the role of treatment facility on patient outcomes.

## Methods

**Figure 1:**
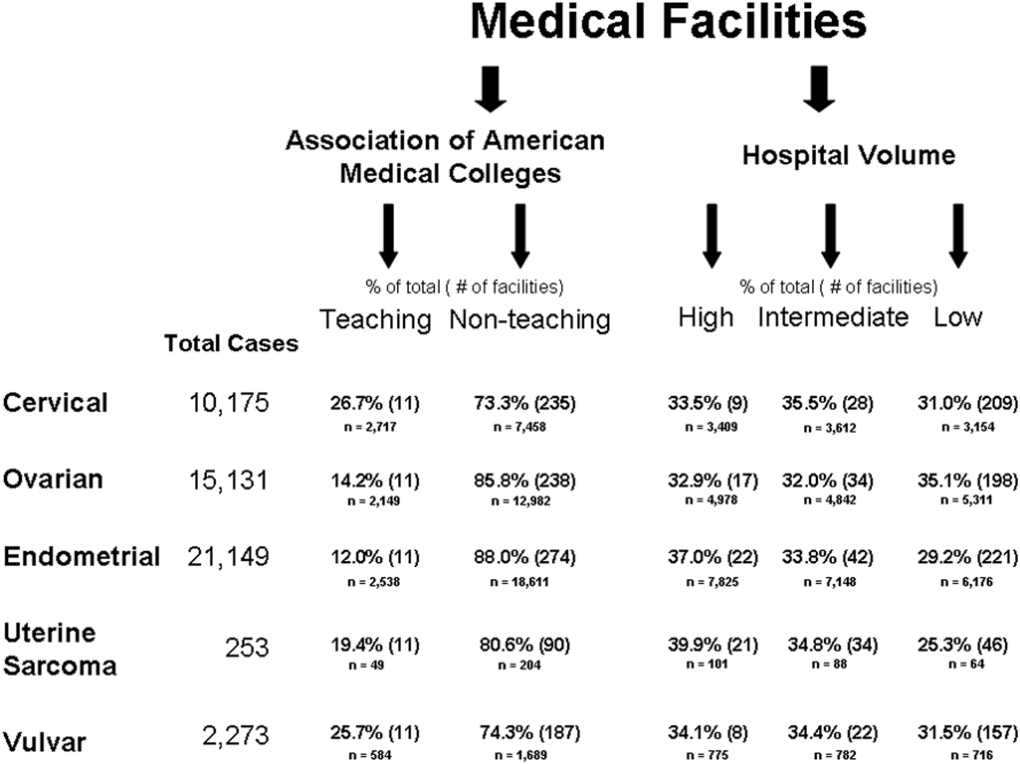
Flow diagram depicting number of incident gynecologic malignancies diagnosed in the state of Florida from 1990–2000.

The 2007 FCDS data set was used to identify all incident cases of cervical, ovarian, endometrial and vulvar malignancies and uterine sarcomas diagnosed in the state of Florida from 1990–2000. A total of 48,981 cases of gynecological cancer were extracted for analysis (Figure 1). Cases with missing information for any key variable, duplicate cases, carcinomas *in situ*, and cases treated by community physicians independent of the hospital or ambulatory care center settings were excluded from the univariate analysis. Incident vulvar carcinoma and uterine sarcoma cases were analyzed; however, case numbers were too small to establish a meaningful interpretation of the univariate five-year survival data. These two gynecological malignancies are described by demographic, social and clinical characteristics, and are included in the multivariate logistic regression analysis, which was performed after combining all types of gynecologic cancers.

Medical facilities were defined as TFs or non-teaching facilities (NTF) based on recognition as a teaching institution by the Association of American Medical Colleges (AAMC). There are currently 11 AAMC-recognized TFs in the state of Florida. The data from FCDS were tabulated to determine the number of treated cancers and surgical resections for five gynecologic cancer types (cervical, ovarian, endometrial, vulvar, and uterine sarcoma) performed at each institution in the state of Florida during the study period.

Medical facilities were grouped into tertiles based on number of surgeries with curative intent performed during the study period. The upper one-third of institutions was classified as HVC, the middle one-third as intermediate-volume centers (IVC), and the lower one-third as low-volume centers (LVC). The definitions of HVC, IVC, and LVC for cervical, ovarian, endometrial and vulvar cancers are shown in Figure 1. For uterine sarcoma, HVCs operated on an average of 5 cases in the 10-year study period, IVCs operated on an average of 3 cases in the 10-year study period, and LVCs operated on an average 1 case in the 10-year study period.

The staging criteria used by the FCDS are consistent with the Surveillance, Epidemiology, and End Result (SEER, National Cancer Institute) summary staging and differ from the International Federation of Gynecology and Obstetrics (FIGO) staging guidelines. In this study, local staging represents disease that does not extend beyond the primary organ, while those having positive lymph nodes at the time of resection were classified as having regional disease. Documentation of distant metastases during the peri-operative period led to classification of affected patients as having distant disease.

Statistical computations were performed with SPSS version 15.0 for univariate analyses and the final multivariate regression was corrected for clustering in facilities using STATA version 8.0. ^[24]^ The chi-square test was used for group comparisons of categorical variables and ANOVA was used for group comparisons of continuous variables. Overall survival was used in lieu of disease-specific survival because the FCDS database contains information only on the primary cause of death. Overall survival was calculated by subtracting the date of death or date of last contact from the time of the initial diagnosis. Site-specific thirty-day and ninety-day survival rates, and five-year survival rates were calculated using the Kaplan-Meier method. Univariate Cox proportional hazards regression was used to identify demographic variables and disease characteristics significantly associated with survival.

Significant variables from the univariate analysis were included in the multivariate regression analysis to determine whether facility characteristics were associated with survival for all gynecologic malignancies. Data on lymph nodes included the number of lymph nodes dissected and whether or not lymph nodes were positive for malignant cells. Due to the co-linearity of these variables, only one of these variables can be entered as a covariate in the final multivariate regression model. We chose to enter the variable depicting number of lymph nodes dissected, as we felt this is a better marker of the extent of surgical intervention undertaken at HVCs and TFs compared to LVCs and NTFs. Tumor histology and stage were covariates entered into the final model to reflect severity of disease.

## Results

### Treatment at a teaching facility versus a non-teaching facility

#### Patient demographic, social, and clinical characteristics

Over the ten-year period studied, 10,175 patients with cervical cancer, 15,131 patients with ovarian cancer, 21,149 patients with endometrial cancer, 2,273 patients with vulvar cancer, and 253 patients with uterine sarcoma were identified. Demographics, social and clinical characteristics of the entire study population treated at TFs and NTFs are summarized in Table 1. The majority of patients in the cohort were Caucasian (n = 43,653, 89.1%) and non-Hispanic (n = 43,901, 89.6%). For cervical, ovarian, and endometrial cancers, patients were treated more frequently at NTFs, and those individuals who were treated at TFs were significantly younger than those treated at NTFs. Regional and distant disease were more commonly treated at teaching facilities, whereas gynecologic cancer treated at non-teaching facilities was more commonly localized disease.

**Table 1:** Demographic, Social, and Clinical Characteristics of the Study Group.

	Cervical								Ovarian								Endometrial							
	overall	Facility			Hospital Volume				overall	Facility			Hospital Volume				overall	Facility			Hospital Volume			
		TF	NTF	*p^a^*	HVC	IVC	LVC	*p^b^*		TF	NTF	*p^a^*	HVC	IVC	LVC	*p^b^*		TF	NTF	*p^a^*	HVC	IVC	LVC	*p^b^*
**Median age at diagnosis (years)**	51.9	48.2	53.2	<0.01	48.7	51.9	55.3	<0.01	64.3	59.3	65.1	<0.01	62.2	64.7	65.8	<0.01	66.7	63.1	67.2	<0.01	65.6	66.9	67.5	<0.01
	**n**	**% of total**							**n**	**% of total**							**n**	**% of total**						
**Overall**	10,175	26.7	73.3		33.5	35.5	31		15,131	14.2	85.8		32.9	32	35.1		21,149	12	88		37	33.8	29.2	
**Age Groups**																								
<40	2,807	33.9	26.3	<0.01	33	27.8	23.8	<0.01	961	9.9	5.8	<0.01	7.1	6	6.1	<0.01	546	4.8	2.3	<0.01	3	2.1	2.7	<0.01
41–65	4,681	51.6	45.6		50.5	48.5	42.2		5,974	52.4	37.7		46.6	38.6	34.6		8,200	50.9	37.3		42.3	38.6	35	
>65	2,426	14.5	28.1		16.5	23.6	34		8,077	37.7	56.5		46.4	55.5	59.3		12,315	44.3	60.4		54.7	59.3	62.3	
Unknown	261								119								88							
**Race**																								
Caucasian	8,043	72.3	82.4	<0.01	77.2	77.7	84.7	<0.01	14,015	89.7	93.6	<0.01	93.8	92.8	92.6	0.11	19,266	85	92.5	<0.01	90.5	92.7	91.7	<0.01
AA	1,911	26.6	16.1		21.8	20.5	14.1		953	9.5	5.8		5.7	6.5	6.8		1,632	14.4	6.9		8.9	6.6	7.7	
Other	138	1.1	1.4		1.1	1.8	1.2		89	0.8	0.6		0.5	0.7	0.6		133	0.6	0.6		0.6	0.6	0.6	
Unknown	83								74								118							
**Ethnicity**																								
Hispanic	1,315	21	10.2	<0.01	18.3	12.3	8.4	<0.01	1,164	13.7	6.8	<0.01	8.6	6.5	8.2	<0.01	1,769	16	7.5	<0.01	12.8	2.6	9.9	<0.01
Non-Hispanic	8,711	79	89.8		81.7	87.7	91.6		13,788	86.3	93.2		91.4	93.5	91.8		19,077	84	92.5		87.2	97.4	90.1	
Unknown	149								179								303							
**Primary Payor**																								
insured	2,682	38.5	52.4	<0.01	42.2	56.2	47.1	<0.01	3,531	42.4	40.6	<0.01	46	38.6	38.3	<0.01	4,677	38.7	39.8	<0.01	42.5	37	39	<0.01
non-insured	780	24.3	10.6		21.6	12.4	9		335	6.8	3.4		4.4	3.6	3.7		438	10.7	2.7		5.4	2.6	2.8	
Medicare	474	4.5	10		5.4	8.8	11.5		1,884	15.3	22.9		23.3	25.6	17.4		2,645	16.2	23.3		24.8	26.8	15.1	
Medicaid	721	21.4	10.2		19	10.9	9.8		309	8.7	2.7		4.6	3.3	3		322	7.4	2.1		2.7	2.8	2.7	
Medicare/Medicaid NOS	825	10.8	16.5		11.4	11.2	22.4		2,568	26.8	30.2		21.6	28.8	37.5		3,705	26.6	32.1		24.3	30.7	40.3	
Government	19	10.8	16.5		0.4	0.5	0.2		11	0.1	0.1		0.3	0.1	0.1		17	0.4	0.1		0.2	0.1	0.1	
Unknown	4,674								6,493								9,345							
**LN examined**																								
none	3,277	26.1	34.4	<0.01	22.9	33.4	40.9	<0.01	5,229	31.9	35	<0.01	29	35.8	38.6	<0.01	7,063	28.5	34.1	<0.01	26.6	34.5	40.7	<0.01
1 through 10	510	5.1	5		6.2	6	2.6		1,165	11.3	7.1		11	7.6	4.8		2,308	14	10.5		13.5	11.5	6.9	
11 through 20	671	8.1	6		10.4	6.8	2.3		423	4.1	2.6		5.2	2.1	1.2		1,128	8.4	4.9		8.9	4.2	2.1	
21–30	537	7.8	4.4		9.5	4.7	1.3		219	1.6	1.4		2.8	1.4	0.2		656	3.5	3		5.6	2.3	0.9	
>30	410	7.3	2.8		6.4	4.4	1		66	0.7	0.4		0.8	0.5	0.1		280	2.1	1.2		2.4	1	0.4	
unknown	4,770								8,029								9,717							
**Tumor stage**																								
Localized	4,663	51.5	55.2	<0.01	54.1	52.3	56.5	<0.01	2,451	17.3	19.6	<0.01	19.2	18	20.5	<0.01	13,849	69.1	76.7	<0.01	73	76.6	78.9	<0.01
Regional	2,993	37.5	33.6		34.7	37.5	30.8		1,492	9.2	12.2		11.9	13.4	10		2,705	17.8	14.4		16.4	14.9	12.3	
Distant	963	11	11.2		11.2	10.1	12.6		8,774	73.5	68.2		68.9	68.6	69.5		1,722	13.1	8.9		10.6	8.5	8.8	
Unknown	1,556								2,414								2,873							
**Tumor Grade**																								
Well differentiated	810	7.2	8.3	<0.01	7.7	7.3	9.1	<0.01	1,062	7.6	6.9	<0.01	8.4	6	6.6	<0.01	7,477	31.8	35.8	<0.01	33.6	36.6	36.2	<0.01
Moderately differentiated	2,473	24.5	24.2		25.5	23.5	23.9		2,208	15.5	14.4		16.4	13.4	14		6,248	28.3	29.7		28.6	30.4	29.8	
Poorly differentiated	2,546	29.5	23.4		29.1	24.7	21		4,574	34.7	29.5		35.2	32.4	23.6		3,590	19.9	16.6		19.1	17.1	14.2	
Undifferentiated	177	1.4	1.9		1.7	1.8	1.7		566	4.5	3.6		3.9	4.1	3.3		411	3.2	1.8		2.6	1.6	1.5	
Unknown	4,169								6,421								3,423							
**Surgical Extirpation**																								
Yes	6,161	62.6	59.9	0.02	68	57.4	56.9	<0.01	11,694	15.3	23.8	<0.01	82.9	78.7	71.1	<0.01	17,993	89.2	84.6	<0.01	89	87.4	77.5	<0.01
No	3,997	37.4	40.1		32	42.6	43.1		3,413	15.3	23.8		17.1	21.3	28.9		3,142	10.8	15.4		11	12.6	22.5	
Unknown	17								24								14							
**Chemotherapy**																								
Yes	1,429	16.1	13.6	<0.01	18.2	13.9	10.3	<0.01	7,593	63.4	50	<0.01	61.2	56.4	38.4	<0.01	1,014	7.9	4.5	<0.01	7.1	4.3	2.6	<0.01
No	8,594	83.9	86.4		81.8	86.1	89.7		7,014	36.6	50	<0.01	38.8	43.6	61.6		19,820	92.1	95.5		92.9	95.7	97.4	
Unknown	152								524								315							
**Radiation**																								
Yes	4,423	45.5	43.6	0.09	42.6	53	35.4	<0.01	450	2.6	3.1	0.25	3.3	3.7	2.1	<0.01	4,523	21.5	21.8	0.67	25.9	26	11.4	<0.01
No	5,597	54.5	56.4		57.4	47	64.6		14,504	97.4	96.9		96.7	96.3	97.9		16,221	78.5	78.2		74.1	74	88.6	
Unknown	155								177								405							

	Uterine Sarcoma								Vulvar							
	overall	Facility			Hospital Volume				overall	Facility			Hospital Volume			
		TF	NTF	*p^a^*	HVC	IVC	LVC	*p^b^*		TF	NTF	*p^a^*	HVC	IVC	LVC	*p^b^*
**Median age at diagnosis (years)**	60	61.3	60	0.77	54.8	58.4	66.4	0.01	67.6	63	69.2	<0.01	64.1	68	71	<0.01
	**n**	**% of total**							**n**	**% of total**						
**Overall**	253	19.4	80.6		39.9	34.8	25.3		2,273	25.7	74.3		34.1	34.4	31.5	
**Age Groups**																
<40	25	13	9.5	0.67	11.6	12.5	4.8	0.04	199	12.3	7.5	<0.01	11.2	7.5	7.4	<0.01
41–65	141	58.7	57		61.1	60.2	47.6		675	37	27.2		35.3	28.1	25.4	
>65	80	28.3	33.5		27.4	27.3	47.6		1,399	50.7	65.3		53.5	64.3	67.2	
Unknown	7								0							
**Race**																
Caucasian	209	69.4	86.6	0.01	81	83.9	85.9	0.63	2,120	90.4	94.3	0.01	91.2	94.5	94.1	0.05
AA	38	26.5	12.4		16	14.9	14.1		125	7.7	4.7		7.5	4.5	4.5	
Other	4	4.1	1		3	1.1	0		28	1.9	1		1.3	1	1.4	
Unknown	2								0							
**Ethnicity**																
Hispanic	30	14.3	11.5	0.63	17.2	9.3	7.8	0.13	139	7.6	5.7	0.11	6.4	8.6	3.4	<0.01
Non-Hispanic	219	85.7	88.5		82.8	90.7	92.2		2,106	92.4	94.3		93.6	91.4	96.6	
Unknown	4								8							
**Primary Payor**																
insured	82	40	57.6	<0.01	50	57.4	54.3	0.14	511	34.3	38	<0.01	31.8	42.5	36.6	<0.01
non-insured	14	17.1	6.8		7.8	9.3	11.4		59	10	2.5		8.1	2.8	1.7	
Medicare	20	11.4	13.6		21.9	9.3	2.9		333	14.3	27.3		25.2	26.2	20.4	
Medicaid	9	20	1.7		4.7	9.3	2.9		74	10.3	3.8		9.1	2.6	4.5	
Medicare/Medicaid NOS	28	11.4	20.3		15.6	14.8	28.6		400	31	28.4		25.8	25.8	36.8	
Government	0	0	0		0	0	0		0	0	0		0	0	0	
Unknown	100								896							
**LN examined**																
none	102	36.7	41.2	0.39	35.6	44.3	42.2	<0.01	759	28.4	35.1	<0.01	25.6	32.9	42.4	<0.01
1 through 10	25	16.3	8.3		13.9	10.2	3.1		244	8.4	11.5		11.9	13.8	6.1	
11 through 20	16	6.1	6.4		12.9	3.4	0		255	16.1	9.5		19.6	10.5	2.9	
21–30	5	0	2.5		4	1.1	0		94	5.1	3.8		7.5	3.5	1.3	
>30	2	2	0.5		0	2.3	0		28	2.9	0.7		2.5	1.2	0	
unknown	103								893							
**Tumor stage**																
Localized	131	47.5	61.5	0.24	56	59.5	63.5	0.77	1,422	68.6	72.7	0.19	67	73.3	75.5	<0.01
Regional	40	25	16.5		19.8	15.2	19.2		476	26.6	23		27.9	23.3	19.7	
Distant	51	27.5	22		24.2	25.3	17.3		88	4.8	4.3		5.1	3.4	4.8	
Unknown	41								287							
**Tumor Grade**																
Well differentiated	67	16.3	28.9	<0.01	28.7	27.3	21.9	0.23	426	36.7	34.4	0.69	34.3	34.6	36.5	0.5
Moderately differentiated	38	8.2	16.7		15.8	14.8	14.1		529	1.2	44.3		44.1	43.4	42.6	
Poorly differentiated	25	14.3	8.8		12.9	8	7.8		247	21.2	20		21.1	20.1	19.4	
Undifferentiated	41	32.7	12.3		19.8	15.9	10.9		15	0.9	1.4		0.4	2	1.5	
Unknown	82	28.6	33.3		22.8	34.1	45.3		1,056							
**Surgical Extirpation**																
Yes	213	87.8	83.3	0.52	90.1	83	76.6	0.06	2,012	91.8	87.4	<0.01	92.8	91	81.3	<0.01
No	40	12.2	16.7		9.9	17	23.4		260	8.2	12.6		7.2	9	18.7	
Unknown	0								1							
**Chemotherapy**																
Yes	27	16.7	9.5	0.19	16	10.5	3.2	0.04	89	3.6	4.1	0.71	4.4	4.3	3.1	0.39
No	221	83.3	90.5		84	89.5	96.8		2,161	96.4	95.9		95.6	95.7	96.9	
Unknown	5								23							
**Radiation**																
Yes	46	20.4	18.1	0.69	19.8	23.5	9.7	0.09	294	11.2	13.8	0.12	12.7	13.6	13	0.88
No	202	79.6	81.9		80.2	76.5	90.3		1,948	88.8	86.2		87.3	86.4	87	
Unknown	5								31							

TF = teaching facility; NTF = non-teaching facility; HVC = high-volume center; IVC = intermediate volume center; LVC = low volume center; AA = African American.

*p^a^* value = chi square analysis of survival at TF vs NTF.

*p^b^* value = chi square analysis of survival at HVC vs. IVC vs. LVC.

#### Survival

**Figure 2:**
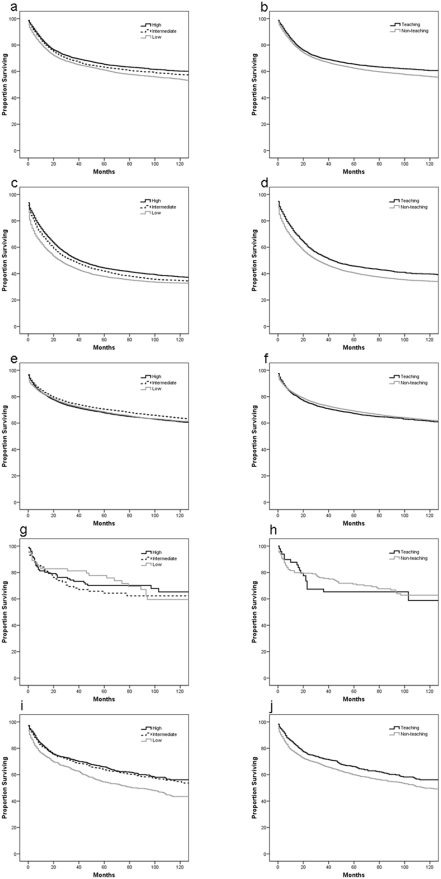
Kaplan-Meier survival curves comparing cumulative survival at teaching facilities (TF) and non-teaching facilities (NTF) for (a) cervical cancer, (b) ovarian cancer, (c) endometrial cancer, (d) uterine sarcoma, and (e) vulvar cancer and at high-volume centers (HVC), intermediate-volume centers (IVC), and low-volume centers (LVC) for (f) cervical cancer, (g) ovarian cancer, (h) endometrial cancer, (i) uterine sarcoma, and (j) vulvar cancer.

Kaplan-Meier plots comparing overall survival at TF and NTF are shown in Figure 2 for (a) cervical cancer, (b) ovarian cancer, (c) endometrial cancer, (d) uterine sarcoma, and (e) vulvar cancer. Five-year survival rates for patients diagnosed with cervical, ovarian and endometrial cancer at TF and NTF are summarized in Table 2. The five-year survival rates for the cohort diagnosed with cervical cancer was 61.7%, for the cohort diagnosed with ovarian cancer was 39.5%, and for the cohort diagnosed with endometrial cancer was 67.3%. As age increased for patients diagnosed with all gynecological cancers, five-year survival rates decreased. For cervical and ovarian cancer, five-year survival rates by univariate analysis were significantly greater for patients treated at TFs compared to those treated at NTFs (63.9% versus 60.9% and 43.9% versus 38.8%; p<0.01, respectively). Among patients diagnosed with cervical cancer, thirty-day and 90-day surgical mortality rates were significantly greater at NTFs compared to TFs (p = 0.04).

**Table 2:** Surgical Mortality and 5-Survival of the Study Group.

	Cervical									Ovarian									Endometrial								
	Overall	*p^a^*	Facility			Hospital Volume				Overall	*p^a^*	Facility			Hospital Volume				Overall	*p^a^*	Facility			Hospital Volume			
			TF	NTF	*p^b^*	HVC	IVC	LVC	*p^c^*			TF	NTF	*p^b^*	HVC	IVC	LVC	*p^c^*			TF	NTF	*p^b^*	HVC	IVC	LVC	*p^c^*
**Surgical Mortality (%)**																											
30 day	1.5		0.8	1.7	0.04	0.9	1.3	2.5	<0.01	6.1		4.5	6.4	0.07	5.0	5.5	7.8	<0.01	3.7		2.0	3.9	0.08	2.9	3.5	5.2	<0.01
90 day	3.7		2.7	4.0		2.9	3.3	5.0		11.6		10.0	11.8		9.9	10.6	14.3		7.2		6.4	7.3		6.9	6.6	8.5	
	**5 year Survival (Proportion Surviving %)**																										
**Overall**	61.7		63.9	60.9	0.00	64.0	61.3	59.5	<0.01	39.5		43.9	38.8	<0.01	42.2	40.4	36.2	<0.01	67.3		65.4	67.5	0.24	66.0	68.9	67.1	<0.01
**Age Groups**																											
<40	76.0	<0.01	73.5	77.1	0.02	75.6	74.3	78.6	0.09	76.6	<0.01	77.4	76.3	0.91	75.9	77.6	76.5	0.98	87.7	<0.01	84.9	88.6	0.43	88.6	88.8	85.5	0.57
41–65	63.1		62.4	63.4	0.58	61.8	62.9	65.0	0.60	47.7		45.4	48.3	0.27	46.6	49.8	47.0	0.13	79.6		74.6	80.5	<0.01	77.2	81.3	81.0	<0.01
>65	41.4		45.7	40.6	0.00	46.5	40.8	39.3	<0.01	28.9		32.4	28.5	<0.01	32.3	29.8	25.6	0.00	58.2		52.8	58.8	0.00	56.2	60.1	58.4	<0.01
**Race**																											
Caucasian	63.2	<0.01	64.6	62.7	0.03	66.1	62.4	61.2	<0.01	39.7	<0.01	44.7	38.9	<0.01	42.2	40.7	36.5	<0.01	69.0	<0.01	68.6	69.0	0.57	67.7	70.5	68.9	<0.01
AA	54.2		61.7	49.8	<0.01	56.9	55.7	47.3	<0.01	33.7		36.4	32.9	0.08	39.6	33.7	29.1	<0.01	46.0		46.4	45.9	0.75	47.3	45.1	45.1	0.57
Other	67.5		59.3	69.9	0.41	58.2	72.2	68.6	0.53	57.1		56.3	57.3	0.93	41.7	70.0	56.4	0.33	74.7		64.3	76.1	0.23	71.1	83.3	68.6	0.42
**Ethnicity**																											
Hispanic	66.4	<0.01	65.3	67.2	0.33	66.8	67.9	63.1	0.18	42.2	0.07	47.0	40.6	0.01	46.9	44.1	36.3	<0.01	66.5	1.00	72.8	64.7	<0.01	66.4	71.4	65.2	0.16
Non-Hispanic	60.9		63.5	60.0	0.00	63.4	60.1	59.2	<0.01	39.3		43.6	38.6	<0.01	41.8	40.3	36.1	<0.01	67.2		64.0	67.6	0.02	65.6	68.7	67.3	<0.01
**Primary Payor**																											
insured	71.6	<0.01	70.1	72.0	0.58	71.1	71.7	72.0	0.92	47.8	<0.01	53.4	46.8	0.00	51.3	47.4	44.6	<0.01	77.8	<0.01	79.3	77.7	0.21	75.8	83.2	75.3	<0.01
non-insured	63.1		64.6	61.9	0.60	65.1	64.7	56.2	0.10	53.5		61.6	51.0	0.09	55.2	52.8	52.5	0.98	71.0		73.2	69.7	0.55	72.8	69.2	68.6	0.54
Medicare	52.0		59.7	50.7	0.10	59.8	53.5	47.1	0.08	37.8		44.6	37.0	0.01	39.5	38.8	34.6	<0.01	63.1		62.6	63.1	0.85	62.1	62.1	66.8	0.28
Medicaid	59.4		60.6	58.4	0.94	59.5	62.7	55.4	0.24	43.4		40.1	45.1	0.62	42.6	42.3	45.3	0.98	65.4		66.8	64.7	0.95	64.8	67.5	63.9	0.77
Medicare/Medicaid NOS	45.1		52.3	43.5	0.05	52.4	42.2	43.1	0.04	31.2		35.3	30.6	0.00	35.4	33.7	27.7	<0.01	58.3		54.3	58.8	0.54	56.5	60.2	58.1	0.13
Government	74.2		100.0	61.9	0.10	80.0	62.5	100.0	0.36	70.0		ND	77.8	0.11	84.6	50.0	ND	0.35	72.4		81.8	66.7	0.79	62.5	80.0	100.0	0.71
**LN examined**																											
none	55.9	<0.01	55.6	56.0	0.37	51.7	56.1	58.2	0.21	31.8	<0.01	37.1	31.0	<0.01	34.0	30.9	31.0	<0.01	65.9	<0.01	63.0	66.2	0.26	63.0	68.8	65.4	<0.01
1 through 10	65.8		63.6	66.6	0.24	60.9	68.4	71.6	0.05	53.7		52.8	54.0	0.87	49.6	56.5	58.7	0.21	71.0		71.2	70.9	0.82	69.8	72.1	71.8	0.55
11 through 20	77.4		74.6	78.8	0.19	76.9	77.9	78.2	0.78	60.7		58.5	61.3	0.57	61.3	64.1	53.0	0.46	73.5		79.0	72.2	0.05	74.0	73.2	71.6	0.65
21–30	80.7		74.9	84.5	0.00	81.2	80.6	77.1	0.82	68.4		78.8	66.3	0.16	71.6	62.8	68.0	0.41	71.0		76.1	70.2	0.21	70.4	71.7	73.1	0.86
>30	82.0		83.1	81.0	0.82	84.9	75.5	93.4	0.05	57.4		63.0	55.8	0.31	60.0	54.5	50.0	0.62	76.6		84.9	74.7	0.06	75.7	75.2	89.5	0.49
Unknown	59.5		62.1	58.6	0.01					40.6		43.8	40.1	0.00	41.8	43.0	37.5	<0.01	66.3		61.1	67.0	<0.01	64.1	67.7	67.3	<0.01
**Tumor stage**																											
Localized	81.0	<0.01	82.7	80.4	0.04	83.0	80.4	79.3	0.02	78.6	<0.01	81.3	78.1	0.10	81.6	78.3	76.2	0.01	77.9	<0.01	78.8	77.8	0.12	77.8	79.1	76.4	<0.01
Regional	48.6		51.2	47.4	0.02	50.5	48.8	45.2	<0.01	48.9		64.0	46.9	<0.01	55.3	46.4	45.0	<0.01	50.3		52.9	49.8	0.07	50.7	51.3	47.8	0.06
Distant	19.5		24.4	17.6	0.00	23.0	17.4	17.6	<0.01	25.8		31.6	24.7	<0.01	27.8	28.0	22.0	<0.01	20.3		19.1	20.6	0.52	17.3	23.2	22.1	0.01
**Tumor Grade**																											
Well differentiated	74.5	<0.01	74.7	74.4	0.85	76.1	72.5	74.9	0.79	68.4	<0.01	64.7	69.0	0.68	71.0	67.7	65.7	0.10	83.0	<0.01	83.1	83.0	0.55	82.4	83.9	82.5	0.25
Moderately differentiated	62.6		66.3	61.3	0.02	65.6	62.0	59.8	0.02	44.7		48.5	44.0	0.08	44.8	46.7	42.7	0.10	71.5		69.0	71.8	0.26	71.8	73.2	69.2	0.01
Poorly differentiated	54.3		59.8	51.9	<0.01	60.7	52.9	46.9	<0.01	31.9		34.9	31.3	0.00	33.6	33.8	27.2	<0.01	44.4		47.6	43.9	0.14	44.7	45.0	43.1	0.18
Undifferentiated	40.5		38.9	41.0	0.63	55.0	32.8	34.6	0.05	32.2		39.5	30.7	0.23	31.4	36.9	27.6	0.01	30.4		30.3	30.5	0.61	25.8	38.2	30.8	0.10
**Surgical Extirpation**																											
Yes	75.0	<0.01	76.5	74.4	0.04	76.5	75.4	72.5	<0.01	45.7	<0.01	47.2	45.4	0.07	46.3	46.6	44.1	<0.01	70.8	<0.01	68.6	71.1	0.08	69.4	72.4	70.6	<0.01
No	41.7		43.2	41.2	0.03	38.5	42.9	42.9	0.03	18.9		26.3	18.2	<0.01	22.8	18.0	17.4	<0.01	48.0		40.2	48.8	0.13	39.3	45.2	55.4	<0.01
**Chemotherapy**																											
Yes	40.5	<0.01	41.0	40.4	0.68	43.7	38.6	37.4	0.49	37.8	<0.01	42.9	36.6	<0.01	40.6	38.0	32.9	<0.01	30.9	<0.01	33.9	30.2	0.43	31.4	29.2	32.7	0.74
No	65.0		68.3	63.9	<0.01	68.5	64.7	62.0	<0.01	41.1		45.5	40.6	<0.01	44.6	42.8	37.9	<0.01	69.3		68.2	69.5	0.68	68.6	70.9	68.4	<0.01
**Radiation**																											
Yes	47.0	<0.01	50.5	45.7	0.00	47.4	48.1	44.6	0.01	40.7	0.29	43.1	40.4	0.74	43.1	41.5	35.8	0.28	60.1	<0.01	61.8	59.9	0.21	59.5	61.8	57.2	0.02
No	73.4		75.1	72.9	0.04	76.4	76.5	68.0	<0.01	39.4		44.0	38.6	<0.01	42.1	40.2	36.1	<0.01	69.5		66.5	69.9	0.04	68.3	71.6	68.7	<0.01

TF = teaching facility; NTF = non-teaching facility; HVC = high-volume center; IVC = intermediate volume center; LVC = low volume center; AA = African American; ND = not determined.

*p^a^* value = log rank analysis of survival between categorical variables.

*p^b^* value = log rank analysis of survival at TF vs NTF.

*p^c^* value = log rank analysis of survival at HVC vs. IVC vs. LVC.

### Treatment at high-volume versus low-volume centers

#### Patient demographic, social, and clinical characteristics

Demographics, social and clinical characteristics of the entire study population treated at HVCs, IVCs, and LVCs are summarized in Table 1. For all types of cancer, individuals treated at HVCs were significantly younger than those treated at IVCs or LVCs. For cervical cancer, IVCs treated more regionally advanced disease compared to HVCs and LVCs, but HVCs tended to treat patients with more poorly differentiated cancer. For ovarian cancer, IVCs treated more regionally advanced disease, but HVCs tended to treat patients with more poorly differentiated cancer. For endometrial and vulvar cancers, HVCs treated more regional and distant disease and tended to treat patient with poorer differentiated cancer. For uterine sarcomas, IVCs treated more distant stage disease compared to HVCs and LVCs, but HVCs tended to treat patients with more poorly differentiated cancer.

#### Survival

Kaplan-Meier plots comparing overall survival at HVCs, IVCs, and LVCs are shown in Figure 2 for (f) cervical cancer, (g) ovarian cancer, (h) endometrial cancer, (i) uterine sarcoma, and (j) vulvar cancer. Five-year survival rates for patients diagnosed with cervical, ovarian and endometrial cancer at HVCs, IVCs and LVCs are summarized in Table 2. For cervical cancer, five-year survival rates were significantly greater for patients treated at HVCs compared to those treated at IVCs or LVCs (64% versus 61.3% versus 59.5%; p<0.01). Thirty-day and ninety-day surgical mortality rates were significantly lower for patients treated at HVCs, compared to IVCs or LVCs (p<0.01).

For ovarian cancer, univariate analysis showed that 5-year survival rates were significantly greater for patients treated at HVCs compared to those treated at IVCs or LVCs (42.2% versus 40.4% versus 36.2%; p<0.01). Thirty-day and 90-day surgical mortality rates were significantly lower for patients treated at HVCs compared to IVCs or LVCs. For endometrial cancer, univariate analysis demonstrated 5-year survival rates were significantly greater for patients treated at IVCs compared to HVCs or LVCs. Thirty-day surgical mortality rates were significantly lower for patients treated at HVCs compared to IVCs or LVCs.

#### Multivariate Analysis

Results of the multivariate analysis including all five gynecologic malignancies using a Cox regression model adjusted for clustering effects are summarized in Table 3. Cox regression models adjusting for clustering effects were also created separately for each malignancy, with no difference in survival seen for patients treated at TFs versus NTFs, or HVCs versus LVCs. An abbreviated regression model for each cancer type is shown in Table 4. TF status and hospital volume status were not significant predictors of survival for gynecologic malignancy. Independent predictors of survival for all gynecologic malignancies studied in the 10-year period were diagnosis of ovarian cancer, age >40 years, African-American race, Medicaid payer status, lymph node examination, tumor stage, tumor grade, surgical extirpation, chemotherapy treatment, and lack of radiation therapy.

**Table 3:** Cox Regression Model for Risk of Death.

	HR	95% CI			*p*
**Cancer**					
Cervical	reference group				
Ovarian	1.13	1.07	-	1.20	<0.01
Endometrial	1.05	0.99	-	1.11	0.13
Uterine sarcoma	0.84	0.61	-	1.16	0.30
Vulvar	ND				
**Hospital Volume**					
High	reference group				
Intermediate+Low	0.96	0.91	-	1.03	0.25
**Facility**					
Teaching	reference group				
Non-Teaching	1.08	0.99	-	1.18	0.08
**Age Groups**					
<40	reference group				
41–65	1.52	1.43	-	1.63	<0.01
>65	2.61	2.41	-	2.83	<0.01
**Race**					
Caucasian	reference group				
AA	1.36	1.27	-	1.45	<0.01
Other	0.84	0.69	-	1.02	0.08
**Ethnicity**					
Hispanic	reference group				
Non-Hispanic	0.99	0.92	-	1.07	0.84
**Primary Payor**					
insured	reference group				
non-insured	1.00	0.91	-	1.11	0.98
Medicare	1.03	0.97	-	1.09	0.39
Medicaid	1.29	1.14	-	1.48	<0.01
Medicare/Medicaid NOS	1.12	1.05	-	1.19	<0.01
Government	0.81	0.47	-	1.39	0.45
Unknown	1.13	1.08	-	1.18	<0.01
**Lymph Nodes Examined**					
none examined	reference group				
1 to 10	0.79	0.73	-	0.85	<0.01
11 to 20	0.67	0.62	-	0.73	<0.01
20 to 30	0.62	0.55	-	0.70	<0.01
>30	0.60	0.49	-	0.75	<0.01
Unknown	0.95	0.92	-	0.99	0.02
**Tumor stage**					
Localized	reference group				
Regional	2.08	1.97	-	2.20	<0.01
Distant	3.82	3.58	-	4.07	<0.01
Unknown	1.67	1.57	-	1.79	<0.01
**Tumor Grade**					
Well differentiated	reference group				
Moderately differentiated	1.47	1.38	-	1.57	<0.01
Poorly differentiated	1.93	1.81	-	2.05	<0.01
Undifferentiated	2.21	2.01	-	2.43	<0.01
Unknown	1.81	1.70	-	1.92	<0.01
**Surgical Extirpation**					
Yes	reference group				
No	1.57	1.52	-	1.64	<0.01
**Chemotherapy**					
Yes	reference group				
No	1.08	1.03	-	1.13	<0.01
**Radiation**					
Yes	reference group				
No	0.90	0.86	-	0.94	<0.01

AA = African American, HR = hazard ration, ND = not determined.

**Table 4:** Cox Regression Model for Risk of Death*.

	Cervical		Ovarian		Endometrial		Uterine sarcoma		Vulvar	
	HR	*p*	HR	*p*	HR	*p*	HR	*p*	HR	*p*
**Hospital Volume**										
High	Reference group		Reference group		Reference group		Reference group		Reference group	
Intermediate	0.89	0.26	0.98	0.69	0.90	0.04	1.33	0.37	1.95	0.08
Low	0.90	0.21	1.01	0.11	0.93	0.16	1.10	0.19	1.23	0.07
**Facility**										
Teaching	Reference group		Reference group		Reference group		Reference group		Reference group	
Non-Teaching	1.15	0.23	1.09	0.12	1.02	0.75	1.58	0.08	1.12	0.44

* regression model includes all covariates - HR shown only for hospital volume and facilty type.

Compared to patients diagnosed with cervical cancer, ovarian cancer patients had a 13% increased risk of death over the 10-year study period. Gynecologic cancer patients aged 41–65 years had a 52% increased risk of death (HR = 1.52, p<0.01) and patients older than 65 years had a 261% increased risk of death (HR = 2.61, p<0.01) compared to patients younger than 40 years. African-American patients diagnosed with gynecologic malignancies were 36% more likely to die (HR = 1.36, p<0.01) in the 10-year study period compared to Caucasian patients. Medicaid patients were 29% more likely to die from a gynecologic malignancy in the 10-year study period (HR = 1.29, p<0.01) compared to privately insured patients. A decreased risk of death from gynecologic malignancy was found when lymph nodes were examined compared to when they were not examined. This effect demonstrated a dose-response relationship, with further risk reduction when more nodes were examined. Patients with gynecological cancer were 2.08 times more likely to die when they had regionally advanced disease (HR = 2.08, p<0.01) and 3.82 times more likely to die when they had distant staged disease (HR = 3.82, p<0.01) compared to patients with localized cancer.

More poorly differentiated gynecological cancers portended a worse prognosis, as an increased risk of death in the 10-year study period was observed in patients with moderately and poorly differentiated malignancies compared to patients with well-differentiated tumors. Patients with a gynecologic malignancy were at a 57% increased risk of death (HR = 1.57, p<0.01) if they were not treated surgically, compared to patients who were treated surgically for their cancers. Those patients who did not receive chemotherapy treatment were at an 8% increased risk of death (HR = 1.08, p<0.01) compared to patients who were treated with chemotherapy. Patients who did not receive radiation therapy for their gynecologic malignancy had a 10% decreased risk of death (HR = 0.90, p<0.01) compared to those patients who were treated with radiation therapy. Stepwise regression models evaluating the impact of patient characteristics, tumor characteristics, and treatment for each individual cancer type did not alter the significance of results (data not shown).

## Discussion

It has been demonstrated that the median survival and cure rates for patients diagnosed with certain malignancies, such as sarcomas of the trunk and retroperitoneum and cancers arising in the esophagus and pancreas, are improved when treatment is provided in specialized centers such as high-volume centers (HVC) and teaching facilities (TF).[5], [19], [25], [26], [27], [28], [29] Herein we have attempted to comprehensively determined the impact of both hospital volume and teaching status for the field of gynecologic malignancies. To our knowledge this represents the first, and largest, study providing an overview of the entire field of gynecological malignancies and the effects of hospital volume and teaching status to date.

The five malignancies studied represent >98% of all gynecologic malignancies reported to the FCDS database. Multiple Cox regression analyses were modeled in order to identify any differences that may have gone undetected. These models included analysis in which each individual cancer was analyzed - cervical, endometrial, and ovarian - and one final model in which all patients with a gynecological malignancy were included. Patients with uterine sarcomas and vulvar cancers were excluded from the individual Cox regression modeling because multivariate analysis of these cancers resulted in no significant predictors of survival for any of the demographic, clinical or treatment variables. We suspect inadequate sample size for these specific cancers as the explanation for these observations.

Overall for all five gynecologic malignancies, and separately for cervical, endometrial, and ovarian cancer, no demonstrable benefit for either high volume center or teaching status on patient survival was observed. We did not have complete data on treating physician specialty, making it difficult to determine what these results mean in terms of specific providers. Of note, we were able to determine that all high volume facilities and teaching hospitals examined in this study had board-certified gynecologic oncologists on staff. Many of the low volume centers did not have board-certified gynecologic oncologists on staff, which suggests that other medical professionals, likely general obstetrician-gynecologists or general surgeons, may have delivered care.

There are several possible explanations as to why we fail to find survival benefits at TFs or HVCs. Gynecologic oncology fellowship training may be well standardized and patients treated by these specialists at NTFs or LVCs are provided equivalent care. In addition, gynecological surgeries - i.e. hysterectomies - are performed for a number of reasons other than for malignancies. General obstetrician-gynecologists and general surgeons are often the providers performing the surgical extirpation for benign reasons. As such, these providers may get the necessary case volume to become proficient with these procedures. Thus, while certain subsets of patients may benefit from treatment by gynecologic oncologists, we did not find evidence for a significant survival advantage for patients when they were treated at TFs or HVCs.

Many studies in ovarian cancer have focused on provider specialty type (general gynecologist versus gynecologic oncologist), rather than volumes, specifically. Several studies have found that treatment at specialized hospitals including high-volume centers with gynecologic oncologists as providers, are more likely to include staging procedures, lymph node biopsy, “optimal” debulking and chemotherapy according to guidelines compared to non-specialized hospitals.[2], [3], [4], [5], [6], [8], [9], [13] Many of these studies were limited by the use of Medicare-linked Surveillance, Epidemiology, and End Results (SEER) data, which includes only patients older than 65 years, a variable we have demonstrated is an independent predictor of worse survival outcomes. Of note, Woodman et al found no survival advantage for ovarian cancer patients treated by high-volume operators compared to low volume-operators; however, they had many fewer cases to analyze than in our cohort.[10]

In simple regression analysis models, common in many outcome studies currently in the literature, independence of each individual patient is assumed. However, outcome studies in which hospital volume or teaching status is examined, patients treated within the same facility are not entirely independent. Outcomes of patients treated at one facility tend to be more similar to one another than the outcomes of patients treated at an entirely different facility, a concept known as clustering.[30] As such, studies that do not account for clustering may exaggerate the statistical significance of differences in outcome by provider.[24], [30]

None of the ovarian cancer volumes studies we could identify in the literature, to date, have accounted for this particular phenomenon. Initially, without correction for clustering, a significant improvement for patients with ovarian cancer treated at teaching facilities was observed in our dataset. We found patients treated at a LVC had an 18% increased risk of death (HR = 1.18, p<0.001). Previous studies on ovarian cancer regionalization have made similar observations. These studies, however, have not included corrections for the clustering phenomenon.[1], [2], [3], [4], [6], [7], [8], [9], [10] After re-analysis, accounting for clustering in our dataset, we no longer find a survival benefit for ovarian cancer patients treated at high-volume centers. This suggests that corrections for clustering is crucial in these types of studies as accurate interpretation of the data may not be possible without it. As such, the proper interpretation of our dataset indicates that current chemotherapy and surgical therapies provided are equivalent at all facilities regardless of teaching status or volume.

Previous studies on endometrial cancer support our findings.^[14,15]^ Hoekstra et al[14] found costs and operative times are increased when general gynecologists participate in the surgical procedure of patients with early stage endometrial cancer, but perioperative outcomes were similar when compared to procedures performed completely by a gynecologic oncologist. In a small tumor registry, Macdonald et al[15] found that disease-free and cause-specific survival were equivalent in patients treated by general gynecologists or gynecologic oncologists for early stage endometrial carcinoma. These studies limited their sample to early stage cancers, while we have demonstrated that independent of stage, treatment at NTFs and LVCs, with presumably fewer gynecologic oncologists, does not confer a survival disadvantage. Based on this data, management of early stage endometrial cancer by a general obstetrician-gynecologist, and perhaps general surgeons, appears reasonable as there is no evidence of better outcomes when treated by a gynecologic oncologist. Diaz-Montes et al[16] found among women ≥80 years of age with endometrial cancer, there was a 62% reduction in the risk of 30-day mortality when they were managed at high-volume hospitals and a 44% reduction in the risk of 30-day mortality when managed by high-volume surgeons. Once again, this study did not account for clustering effects, which may explain our different results.

The FCDS, which currently includes over 2.7 million records, is a population-based registry of all cancer cases diagnosed and treated in the state of Florida, which represents about 6% of the total U.S. population. Although it represents an excellent database for comparative outcomes analysis, it is not without limitations. This includes the lack of information in the registry on household income; however, information on insurance status and race may serve as an adequate proxy for socioeconomic status. Our dataset also did not contain information on co-morbidities; therefore, the data presented reflects overall survival and not cause-specific survival. Others have demonstrated that inclusion of co-morbidities may not significantly affect results. Eisenkop et al[4] studied the impact of subspecialty training in gynecologic oncology on the management of advanced ovarian cancer and reported that the distribution of perioperative morbidity among ovarian cancer patients treated by subspecialists versus general obstetrician-gynecologists was not significantly different between the two groups. Furthermore, we submit that ambulatory patients with a large number of comorbidities are less likely to travel farther to regionalized centers.[18] Thus, TFs or HVCs may not necessarily have seen sicker patients and the lack of survival advantage seen at these facilities may not be related to the overall health of the patient. Given the fact that we saw no survival advantage in patients receiving regionalized care after controlling for clustering, it is likely that comorbidity data would not have altered our results. Finally, follow-up of patients in the FCDS is passive and determined generally by report of death certificate to the social security data set. This may result in under-estimation of patient deaths by up to 5%.[5], [19], [25], [26], [27], [28], [29]

Studies on cancer treatment in the surgical literature suggest certain cancers should be treated at HVCs or TFs.[5], [19], [25], [26], [27], [28], [29] Such studies have resulted in a number of national initiatives to improve the delivery of cancer care. The American College of Surgeons, through the development of the National Surgical Quality Improvement Program (NSQIP), has demonstrated since 1991 that the systematic collection, analysis and feedback of risk-adjusted surgical data, including that on hospital volumes, leads to improved outcomes. Although we were not able to demonstrate improved survival outcomes for gynecologic malignancies through regionalized care, the NSQIP initiative of measuring hospitals surgical outcomes and identifying deficiencies can be used as a model for gynecologic malignancies as some differences in regionalized care may exist. Further studies on regionalization of care may serve to improve survival outcomes, just as NSQIP has attempted to do for general surgery and its subspecialties.

In conclusion, we noted improved short-term (30-day and 90-day) survival for cervical, ovarian and endometrial cancers treated at HVCs compared to LVCs, but not long-term (overall) survival for patients with gynecologic malignancies treated at HVCs. After adjusting for clustering effects, there is not a long-term survival advantage for gynecologic cancer patients treated at a TFs or HVCs. These findings suggests further regionalization of gynecologic cancer care will not improve overall patient outcomes.
